# reDA: differential abundance testing on scATAC-seq data using random walk with restart

**DOI:** 10.1093/bioinformatics/btaf459

**Published:** 2025-08-29

**Authors:** Zirui Chen, Jiao Hua, Lu Ba, Tianyun He, Boran Yang, Jing Qi, Shuilin Jin

**Affiliations:** School of Mathematics, Harbin Institute of Technology, Harbin, 150000, China; Zhengzhou Research Institute, Harbin Institute of Technology, Zhengzhou, 450000, China; School of Mathematics, Harbin Institute of Technology, Harbin, 150000, China; Zhengzhou Research Institute, Harbin Institute of Technology, Zhengzhou, 450000, China; School of Mathematics, Harbin Institute of Technology, Harbin, 150000, China; School of Mathematics, Harbin Institute of Technology, Harbin, 150000, China; Zhengzhou Research Institute, Harbin Institute of Technology, Zhengzhou, 450000, China; School of Mathematics, Harbin Institute of Technology, Harbin, 150000, China; Zhengzhou Research Institute, Harbin Institute of Technology, Zhengzhou, 450000, China; School of Mathematics, Harbin Institute of Technology, Harbin, 150000, China; Zhengzhou Research Institute, Harbin Institute of Technology, Zhengzhou, 450000, China; School of Mathematics, Harbin Institute of Technology, Harbin, 150000, China; Zhengzhou Research Institute, Harbin Institute of Technology, Zhengzhou, 450000, China

## Abstract

**Summary:**

Identifying cell states associated with disease progression or experimental perturbations from single-cell Assay for Transposase Accessible Chromatin using sequencing (scATAC-seq) data is critical for unraveling disease pathogenesis. However, the high dimensionality, extreme sparsity, and nearly binary nature of scATAC-seq data pose significant challenges. Here, we present reDA, a cluster-free computational framework that performs differential abundance testing based on the random walk with restart. Through comprehensive experiments on simulated and real datasets, reDA outperforms six baseline methods, demonstrating superior accuracy, computational efficiency, and the ability to capture disease-specific molecular signatures.

**Availability and implementation:**

The reDA along with detailed documentation is freely available at https://github.com/Jinsl-lab/reDA. It can be seamlessly integrated into existing scATAC-seq analysis workflows.

## 1 Introduction

Single-cell technologies have revolutionized our understanding of biology, from interrogating cellular heterogeneity to identifying disease-specific processes (Olsen and Baryawno 2018). Single-cell Assay for Transposase Accessible Chromatin using sequencing (scATAC-seq) measures the open chromatin status of individual cells (Buenrostro *et al.* 2015), providing insight into gene regulation and epigenetic heterogeneity at single-cell resolution (Cusanovich *et al.* 2018, Ranzoni *et al.* 2021). Exponential increases in the scale and availability of scATAC-seq are now triggering a comprehensive characterization of the dynamic changes of cells in different biological states, especially cell state transitions in disease progression, developmental processes, and experimental perturbations (such as drug treatment, gene knockout) (Domcke *et al.* 2020, Aissa *et al.* 2021, Morabito *et al.* 2021). Cell state refers to the changes and variations in cell phenotype or function under different conditions or stimuli (Rukhlenko *et al.* 2022, Rafelski and Theriot 2024). The common analysis is to detect perturbed cell states by quantifying changes in the abundance of predefined cell clusters under different conditions or perturbations (Stuart *et al.* 2021, Heumos *et al.* 2023). However, this strategy assumes that the underlying biology is well captured by the imposed structure and relies heavily on clustering analysis, hindering the discovery of some rare cell subpopulations.

Several clustering-independent methods have been designed for single-cell RNA-seq (scRNA-seq) data, such as Cydar (Lun *et al.* 2017), DA-seq (Zhao *et al.* 2021), MELD (Burkhardt *et al.* 2021), CNA (Reshef *et al.* 2022), Milo (Dann *et al.* 2022), and PENCIL (Ren *et al.* 2023), offering the more granular approach for identifying condition-associated cells (Yi *et al.* 2024). Most of these methods estimate cell abundance based on the nearest neighbor graph, focusing on the local or global information of cell neighbors. Moreover, as they are specifically developed for scRNA-seq data, their applicability to scATAC-seq remains limited. Unlike scRNA-seq data, scATAC-seq measures DNA accessibility, but suffers from inherent data extremely sparsity and binarization due to low copy numbers, typically detecting only 1%–10% of accessible regions per cell, which significantly complicates biologically relevant feature extraction, impairs accurate intercellular variation assessment (Chen *et al.* 2019). Furthermore, the original features of scATAC-seq are open regions on the genome, usually up to hundreds of thousands, which greatly increases the computational complexity. Therefore, there is an urgent need to develop methods for scATAC-seq data to accurately identify and quantify cell states in response to changing conditions such as disease progression, developmental processes, and experimental perturbations from heterogeneous cell populations.

To address this gap, we developed reDA, a differential abundance test framework based on random walk with restart, for scATAC-seq data. To better measure the abundance of cells under different conditions, reDA introduces a random walk with restart, which can better capture the local and global information of the shared nearest neighbor (SNN) graph, mitigate the effects of information loss and information redundancy (Cowen *et al.* 2017, Zhou *et al.* 2019), and thus reliably identify condition-specific cell states based on association tests. Through comprehensive experiments on multiple simulated and real scATAC-seq datasets, we demonstrated that reDA outperforms six state-of-the-art methods in inferring condition-related cell states. In addition, we further demonstrated on colorectal cancer (CRC) data that reDA can accurately identify CRC-related cell subpopulations and further discovered transcription factor motifs related to CRC, indicating that reDA has important guiding significance in revealing the pathogenesis of the disease. Finally, we tested the running time consumption of reDA, which can process 100 000 cells in 3 min and outperforms most other existing methods.

## 2 Materials and methods

**Figure 1.**
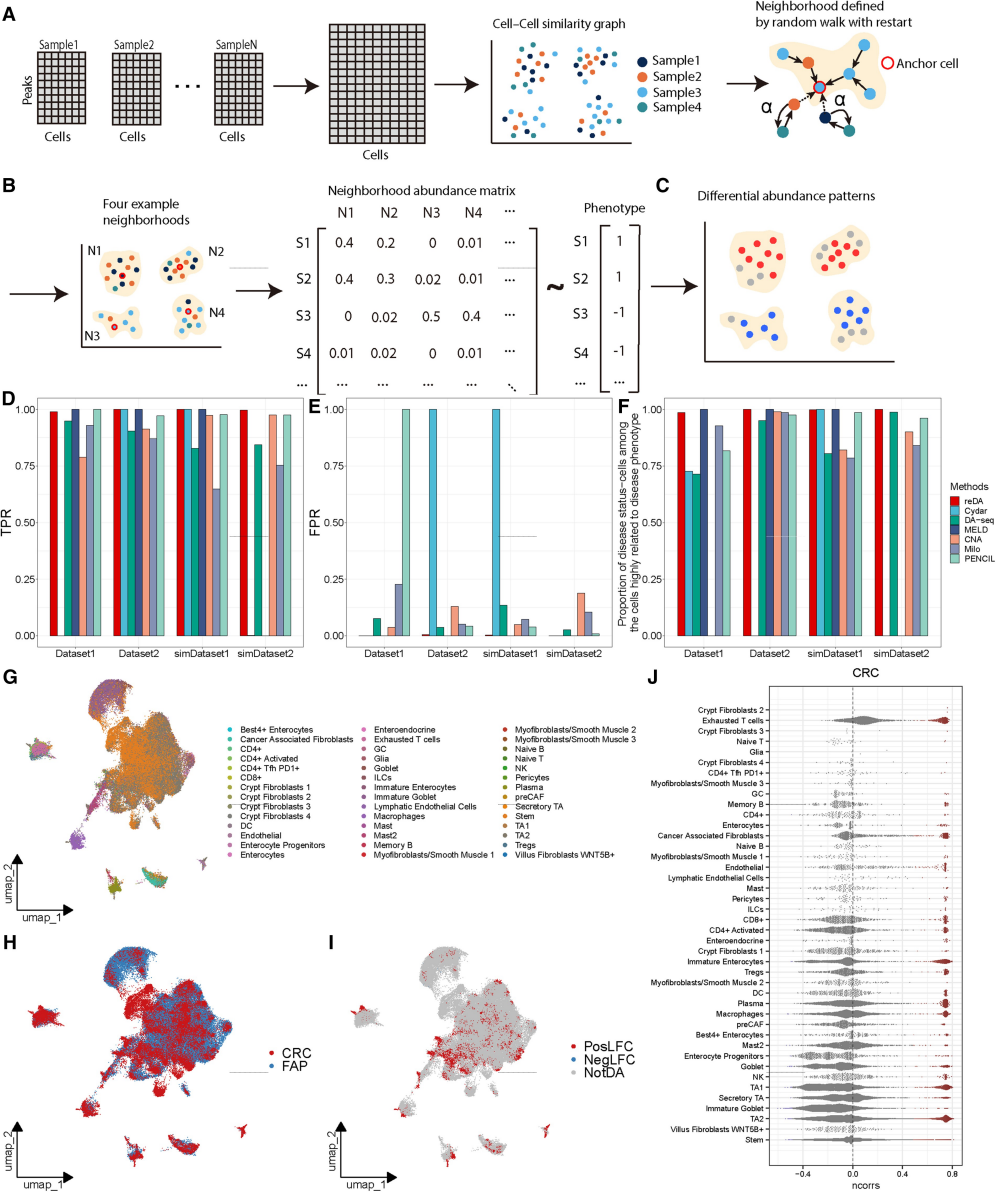
The workflow of reDA and its performance. (A) reDA constructs a cell–cell similarity network in a multi-sample scATAC-seq dataset and defines a neighborhood for each cell. (B) NAM is constructed according to the neighborhood defined by random walk with restart. (C) Phenotype-related cell subsets were obtained by association testing of NAM. (D, E) TPR and FPR of reDA and comparison methods on real and simulated data with ground truth labels. (F) Proportion of disease status-cells among the cells highly related to disease phenotype identified by the method. (G, H) UMAP embedding of 98 747 cells from FAP and CRC patients. Cells are colored by cell types (G) and sample conditions (H). (I) Cell subsets highly associated with CRC identified by reDA. (J) Beeswarm plot showing the distribution of neighborhood coefficients from different cell types. Differential abundance cells at FDR 5% are colored.

reDA detects the cell subsets that are differentially abundant between conditions (phenotypes) (Fig. 1A–C). First, reDA constructs a neighbor graph of scATAC-seq from multiple samples. Then, the neighborhood abundance matrix (NAM) is calculated for each cell based on the random walk with restart. Finally, an association test framework is introduced to combine the condition information of the sample and the NAM matrix for global and local association tests. This allows to detect regions with different abundance, helping to pinpoint cell subpopulations that are highly correlated with clinical phenotypes.

### 2.1 Cell-to-cell similarity network construction

The reDA takes the peak-by-cell count matrix of scATAC-seq as input data, where there are M cells from N samples. We use the SNN algorithm to construct the M×M cell–cell similarity network graph A after preprocessing, latent semantic indexing (LSI) dimensionality reduction, and batch correction on the count matrix (Text S1, available as supplementary data at *Bioinformatics* online).

### 2.2 Construction of NAM

reDA uses a random walk with restart to construct neighborhood abundance matrix. Firstly, we calculate a M×M walking matrix W. $W_{i,i^{,}}$ represents the probability of moving one step from cell i to cell $i^{,}$. This is given by


$$W=D^{-1}A$$

where D is the diagonal matrix of the sum of the edge weights of each cell. Here, reDA introduces a non-zero restart probability α, which represents the probability of each walk back to the starting cell. The probability of a random walk from cell i to cell $i^{,}$ after s steps is represented as the degree to which cell i belongs to the neighborhood of cell $i^{,}$. Specifically, the random walk with restart is defined as:


$$r^{s}=(1-\alpha )Wr^{s-1}+\alpha I$$

where $I\in R^{M\times M}$ is the identity matrix.

After defining the neighborhood, we construct NAM. The probability that the cells from sample n reach the neighborhood of cell i through s steps is defined as


$$R_{n,i}≔\Sigma_{i^{,}\in C(n)}r_{i^{,},i}^{s}.$$

Finally, NAM is obtained by normalizing R:


$$Q_{n,i}=\frac{R_{n,i}}{\Sigma_{i^{,}}R_{n,i^{,}}}.$$

Regarding the choice of step size s, we follow the principle of minimizing the neighborhood size to preserve the informative granularity while ensuring that neighborhoods are not dominated by cells from a few samples (Reshef *et al.* 2022). So, we introduce the indicator from CNA, which is quantified by measuring the kurtosis of each column of the NAM. With the increase of the walking step size, kurtosis decreases and the situation that a few samples dominate the neighborhood is improved. Therefore, we set the neighborhood size to be balanced when the median kurtosis across neighborhoods decreases by <3 or the median kurtosis across neighborhoods is <8. Notably, we found that reDA performs robustly when the restart probability is set to 0.3, and even without the restart mechanism, it still outperforms CNA (Figs S1, S2, and S14, available as supplementary data at *Bioinformatics* online). However, if the restart probability continues to increase, it constrains the search capability of the random walk process for neighborhood cells. This limitation fails to mitigate the dominance of a few sample cells within the neighborhood, leading to persistently high kurtosis levels, hindering the convergence of iteration, and results in a significant decline in model performance.

### 2.3 Testing for differential abundance

To test differential abundance, we adopt the association test framework from CNA. First, based on the NAM matrix, PCA is applied to control for sample covariates. A global association test is then performed to link the clinical phenotype to the variation between samples, followed by the local test was used to detect the association between neighborhoods and the phenotype, which produces the neighborhood coefficients that represent the correlation between neighborhoods and clinical phenotypes. Finally, we evaluate the statistical significance of each neighborhood coefficient and estimate the empirical false discovery rate (FDR). Further details of the statistical framework are provided in Text S2, available as supplementary data at *Bioinformatics* online.

### 2.4 Data collection and pre-processing

*Real datasets.* We use the scATAC-seq dataset from GSE201336. A total of 98 747 cells was selected from 14 familial adenomatous polyposis (FAP) and 4 CRC samples as Dataset1. Also, 10 FAP samples and 9 healthy samples were selected, a total of 83 694 cells as Dataset2 (Text S1, available as supplementary data at *Bioinformatics* online, Table S1, available as supplementary data at *Bioinformatics* online).

*Simulated datasets.* We used the simATAC (Navidi *et al.* 2021) tool to generate the simulation data (Text S1, available as supplementary data at *Bioinformatics* online, Table S1, available as supplementary data at *Bioinformatics* online). The simDataset1, 3 and 5 were generated by GSE192838, and both simDataset2 and 4 were generated by GSE181062. To identify the optimal restart probability, similarly, we generated additional simulated datasets (simDataset6-10) with varying cell numbers.

*Data pre-processing.* We pre-processed the scATAC-seq data using Signac pipeline. Firstly, built a unified peak set by overlapping all peaks and generated count matrix for each dataset based on this unified peak set, and created Seurat objects which were subsequently merged. Next, we ran the LSI to reduce dimensionality. Furthermore, we corrected batch effect and constructed the nearest neighbor graph. The dimensions 2–30 were used as input.

## 3 Results

To assess the capability and accuracy of reDA, reDA was benchmarked against six state-of-the-art methods, including CNA, Milo, DA-seq, MELD, Cydar, and PENCIL, on two real datasets and two simulated datasets. Although these six existing methods were not originally designed for scATAC-seq data, they can be adapted to it through additional processing (Text S3, available as supplementary data at *Bioinformatics* online). After generating ground truth labels for each dataset (Figs S3A, S4A, S5A, and S6A, available as supplementary data at *Bioinformatics* online), we used true positive rate (TPR) and false positive rate (FPR) to quantify the accuracy of these methods (Text S5, available as supplementary data at *Bioinformatics* online). The results showed that the reDA outperforms existing methods (Fig. 1D and E, Figs S3–S6, available as supplementary data at *Bioinformatics* online, Tables S2 and S3, available as supplementary data at *Bioinformatics* online), demonstrating the highest TPR and the lowest FPR. It suggested that reDA detected the simulated differential abundance regions with high sensitivity.

Next, we tested the ability of reDA and other methods to identify cell subpopulations associated with disease phenotypes on these four datasets. The results indicated that the proportion of disease status-cells among cells identified by reDA was higher than that of other methods (Fig. 1F–I, Figs S7, S8A–I, available as supplementary data at *Bioinformatics* online, Table S4, available as supplementary data at *Bioinformatics* online). In real datasets from FAP tissue and CRC tissue samples, we found that cell states associated with cancer phenotype identified by reDA mainly include regulatory T cells (Tregs), exhaust T cells, enterocytes, and cancer-associated fibroblasts (CAFs), as observed from the fact that differential abundance cells are enriched in these cell states (Fig. 1J). In the datasets from FAP tissue and healthy colon tissue samples, reDA identified cell states associated with the polyp phenotype, including goblet cells, enterocytes, and immature goblet cells (Fig. S8J, available as supplementary data at *Bioinformatics* online). These findings are consistent with published evidence that these cell states are highly correlated with the progression of CRC malignant tumor continuum (Becker *et al.* 2022), which indicates that reDA identifies cell subsets that play a key role in cancer progression.

In order to prove the ability of reDA to improve the downstream analysis efficiency, we performed motif analysis on the differentially accessible peaks of cell subsets highly related to CRC identified in CRC tissue and FAP tissue samples to find disease-related motifs (Text S4, available as supplementary data at *Bioinformatics* online, Fig. S9, available as supplementary data at *Bioinformatics* online). Among the top 30 motifs enriched in these cell subsets, we found KLF15, KLF16, SP1, SP3, EGR1, ZNF148, and TFDP1 (Fig. S10, available as supplementary data at *Bioinformatics* online). KLF15 has been found to inhibit the development of CRC (Cao *et al.* 2024), while EGR1 can promote the apoptosis of CRC cells (Lee *et al.* 2008). Recent research showed that TFDP1 was identified as a critical CRC molecule (Song and Ma 2023). The high expression of ZNF148 is negatively correlated with the malignant phenotype of CRC patients, which may be an important prognostic factor for patients after surgery (Gao *et al.* 2013). KLF16, SP1, and SP3 promote the development of CRC (Liu *et al.* 2021, Ma *et al.* 2022). Meanwhile, we analyzed the background cells of the four groups of cell subsets as a negative control (Fig. S11, available as supplementary data at *Bioinformatics* online). We found that there were significant differences in motifs between the experimental group and the control group, indicating that non-differentially abundant cell populations do not exhibit similar motifs. In summary, these motifs are highly correlated with CRC, demonstrating the ability of reDA to identify disease-associated cell states. Further, we performed the overlap analysis between differentially accessible regions (DARs) of disease-associated cell subsets and CRC-associated genetic loci identified by GWAS. The observed overlaps with known risk loci suggest that the identified chromatin accessibility changes are potentially associated with disease-related genetic variation, providing additional evidence for the reliability and effectiveness of the reDA (Text S7, available as supplementary data at *Bioinformatics* online).

Additionally, we evaluated the operational efficiency of reDA, and compared it with six methods (Fig. S12, available as supplementary data at *Bioinformatics* online). We used simATAC to simulate seven single-cell datasets with continuously increasing cell numbers, and made use of time function in R and Python to record the start and end system time of the key steps. reDA takes less time than most methods on the seven datasets and is only slightly higher than CNA. Among them, reDA is significantly lower than Milo and Cydar on all seven datasets, but has better recognition performance in previous experiments. It can be seen that reDA does not improve recognition accuracy by sacrificing operational efficiency.

Finally, to further evaluate the robustness of reDA in the presence of technical variability, we introduced artificial batch effects of varying magnitudes into the five datasets (Texts S1 and S6, available as supplementary data at *Bioinformatics* online), following strategies adopted in previous studies (Dann *et al.* 2022, Yi *et al.* 2024). We then assessed the performance of reDA and competing methods under these conditions (Fig. S13, available as supplementary data at *Bioinformatics* online). The results indicate that reDA remains robust against batch effects, basically achieving higher TPR and lower FPR compared to baseline methods.

## 4 Conclusion

We present reDA, a flexible and time-efficient differential abundance testing tool based on the random walk with restart, specifically designed for large-scale scATAC-seq data analysis. Compared with existing approaches such as CNA, which was originally developed for scRNA-seq data, reDA is specifically tailored for scATAC-seq data and addresses its unique characteristics such as extreme sparsity and near-binary profiles. By introducing a random walk with restart model and adaptive termination criteria during neighborhood definition, reDA achieves more robust and accurate differential abundance testing.reDA can be used to study different sample phenotypic attributes, thereby improving the understanding of disease pathology, risk, and treatment. reDA could be seamlessly integrated with existing workflows and will provide valuable assistance for the study of cellular epigenomic landscapes.

Despite its promising performance, reDA has certain limitations. While it allows adjustment for additional conditions as covariates, it is currently unable to simultaneously model multiple conditions or phenotypes as outcomes, which may limit its ability to capture the complex relationships between different conditions in some biological scenarios. Furthermore, reDA is presently designed for scATAC-seq data, and future efforts will focus on extending it to single-cell multi-omics data, enabling more comprehensive insights into gene regulation across cellular contexts. An additional promising direction involves incorporating prior biological knowledge (e.g. established regulatory networks or cell lineage hierarchies) to enhance both the interpretability and precision of the analytical results.

## Supplementary Material

btaf459_Supplementary_Data

## Data Availability

The data underlying this article are available in the article and in its online supplementary material. No new data were generated in support of this research. The data and code files used in the study can be found at https://doi.org/10.5281/zenodo.15805483.
