# Supplementary material for: reDA: differential abundance testing on scATAC-seq data using random walk with restart
Source: Bioinformatics. 2025 Aug 29;41(10):btaf459. doi: 10.1093/bioinformatics/btaf459 (PMC12553332; doi:10.1093/bioinformatics/btaf459)
Supplement: btaf459_Supplementary_Data [file btaf459_supplementary_data.zip › Supplementary Information.pdf]

Supplementary Information

reDA: differential abundance testing on scATAC-seq data using random walk with restart

Zirui Chen[1,2,†], Jiao Hua[1,2,†], Lu Ba[1,†],Tianyun He[1,2], Boran Yang[1,2,], Jing Qi[1,2,*], Shuilin Jin[1,2,*]

[1]School of Mathematics, Harbin Institute of Technology, Harbin 150000, China

[2]Zhengzhou Research Institute, Harbin Institute of Technology, Zhengzhou 450000, China

[*]To whom correspondence should be addressed. E-mail(s): jinsl@hit.edu.cn, qijing@hit.edu.cn.

[†]The authors wish it to be known that, in their opinion, the first three authors should be regarded as Joint First Authors.

Supplementary Texts

Text S1. Data collection and pre-processing

Real datasets. We use the scATAC-seq dataset from GSE201336. In order to study the changes in the phenotypic continuum from healthy colon to colorectal cancer (CRC), we designed two sets of data. Firstly, we selected a total of 98747 cells from 14 familial adenomatous polyposis (FAP) samples and 4 CRC samples as Dataset1 to study the transformation process from polyps to cancer. In addition, we selected 10 FAP samples and 9 healthy samples, a total of 83694 cells as Dataset2, to study the transformation process from normal tissue to polyps.

The above dataset contains cell types that are highly related to the phenotypic transformation process: stem cells, enterocytes, immature goblet cells, goblet cells, regulatory T cells (Tregs), exhausted T cells, pre-cancer-associated fibroblasts (preCAFs), cancer-associated fibroblasts (CAFs).

Simulated datasets. To evaluate the identification accuracy and stability of the proposed method, we generated simulated count matrices (bin × cell) using the simATACSimulate() function from the R package simATAC, based on two scATAC-seq datasets. Firstly, we used a human occipital cortex sample with Clonal hematopoiesis of indeterminate potential (CHIP) and a human occipital cortex sample without CHIP from GSE192838 to simulate 10 groups of diseased samples and 10 groups of healthy samples, a total of 20,871 cells as simDataset1. The other group of data used a renal tumor sample and an adjacent normal tissue sample from GSE181062 to simulate 10 groups of diseased samples and 10 groups of healthy samples, a total of 20871 cells as simDataset2. In order to compare the running speed, we also used the simATACSimulate() function to simulate the scATAC-seq dataset with increasing number of cells (5k, 10k, 15k, 30k, 50k, 80k, 100k). To validate the performance of reDA in the presence of batch effects, we introduced three additional simulated datasets. Both simDataset3 and simDataset5 were generated using samples from GSE192838, creating simulated datasets with equal numbers of disease and healthy samples. Specifically, simDataset3 consists of 8 groups with 40,101 cells, while simDataset5 consists of 10 groups with 50,102 cells. Similarly, simDataset4 was generated using samples from GSE181062, producing a simulated dataset with 8 groups and 40,101 cells, where the number of disease samples is equal to the number of healthy samples. To identify the optimal restart probability for our model, we followed the aforementioned pipeline to generate additional simulated datasets (simDataset6-10) with varying cell numbers.

Data pre-processing. We pre-processed the scATAC-seq data using Signac pipeline. Since there were different peak sets between datasets, we first built a unified peak set by merging all overlapping peaks using the Reduce() function. Based on this unified peak set, we generated peak-by-cell count matrix for each dataset using the FeatureMatrix() function and created Seurat objects with default parameters, which were subsequently merged using the Merge() function. Next, we normalized the scATAC-seq data using the RunTFIDF() function. Then, we identified top features (min.cutoff = 20) and ran singular value decomposition (SVD) on the TD-IDF matrix to reduce dimensionality via RunSVD() function. Furthermore, we used

RunHarmony() function to correct batch effect according to the sample and constructed a shared nearest neighbor (SNN) graph using the FindNeighbors() function (dims=2:30). The nonlinear dimensionality reduction (UMAP) was generated via the RunUMAP() function. The dimensions 2 to 30 were used as input for the algorithm.

Text S2. Association test

In order to test the differential abundance, we adopt the association test framework introduced by CNA. First, the PCA was performed on NAM

$$\bar{Q} = UDV^T.$$

where $\bar{Q}$ is column-standardized NAM. U is a $N \times d$ matrix, D is a $d \times d$ matrix, and V is $d \times M$ matrix.

Next, a global association test links the clinical phenotype with the variation between samples, and a local association test detects the association between the neighborhood and the clinical phenotype. These are given by

$$y = U^k \beta^k + \epsilon,$$
$$\gamma := V^k D^k \beta^k.$$

where y is the samples attribute vector, U^k is the first k column of U, β^k is a coefficient vector, and ϵ is mean-zero noise. The V^k is the first k column of V and D^k is a $k \times k$ upper-left submatrix of D. The γ is referred as the neighborhood coefficients of length M, representing the correlation between neighborhood and clinical phenotype. Several values of k are chosen based on sample number, and the k with the minimal P value in multivariate F-test for the null hypothesis $H_0\colon \beta^k = 0$ is selected. Finally, the statistical significance is evaluated by comparing the number of γ values above a threshold to the average in null versions, estimating the empirical FDR.

Text S3. Benchmarking reDA against existing methods

We benchmarked the performance of reDA against six existing methods, including Cydar, DA-seq, MELD, CNA, Milo, and PENCIL. Since these existing methods were developed for scRNA-seq data, in order to ensure a more rigorous comparison between reDA and the six existing methods, we additionally processed the inputs of these six comparison methods to make them applicable to scATAC-seq data. Specifically, we processed the scATAC-seq count matrix into the low-dimensional embedding matrix according to the Signac pipeline, which is an analysis pipeline designed for scATAC-seq data, and used this embedding matrix as the input of these six existing methods to compare with reDA. In this way, we minimized the impact of omics data differences on the results of these six existing methods, thereby ensuring a fair and scientific comparison.

1. Cydar: We use Cydar by assigning cells to high-dimensional hyperspheres and calculating the difference in their cell numbers to quantify the abundance changes of cells under different conditions. We took the embedding matrix without batch effect as input and used the functions implemented in https://github.com/CompCy-lab/benchmarkDA.
2. DA-seq: DA-seq evaluates the differential abundance scores of cells under different conditions based on k-nearest neighbor across different scales and logistic regression models. We took the embedding matrix without batch effect as input and used the tutorials implemented in https://github.com/KlugerLab/DAseq.
3. MELD: MELD uses the kernel density estimate of the graph to calculate the conditional attribution probability of each cell. As recommended by the authors, we didn't correct batch effect and took the embedding matrix as input. We used the tutorials implemented in https://github.com/KrishnaswamyLab/MELD. For the real phenotype experiment, due to the different number of samples in the control group and the experimental group, we divided the samples into two groups according to the phenotype.
4. CNA: CNA proposes a covariable neighborhood method to capture areas with non-uniform mixing of conditional labels. Since the structure of CNA is similar to reDA, we generated a cell-cell similarity matrix as input according to the reDA analysis process.
5. Milo: Milo analyzes the abundance differences of the neighborhood graph through k-nearest neighbor and negative binomial generalized linear models. We took the embedding matrix without batch effect as input and used the tutorials implemented in https://github.com/MarioniLab/miloR.
6. PENCIL: PENCIL identifies high-confidence subpopulations through a rejection learning strategy. We took the embedding matrix without batch effect as input and used the tutorials implemented in https://github.com/cliffren/PENCIL.

Text S4. Motif analysis

We performed motif analysis in Signac. Firstly, we ran the AddMotifs() function to add the motif information required for motif analysis. Then, we used the FindMarkers() function to find differentially accessible peaks between target cell subset and all other cells. Finally, we made use of FindMotifs() function to perform a hypergeometric test and find overrepresented motifs.

Text S5. Evaluation metrics

Definition of ground truth. For all simulated and real datasets, we generate ground truth for each cell. Firstly, we select the target group based on the phenotype and generate the probability according to the process in Milo. Then, we generate the condition label (C1 or C2) for each cell according to the probability and assign them to 16 simulation samples. Finally, according to the percentage of the target population to the total number of cells ($tPor$), we select the cells with the highest probability of $tPor$ in C2 as PosLFC and the cells with the lowest probability of $tPor$ as NegLFC.

Evaluation. In order to evaluate the recognition effect of the method on disease-related cell subsets, we select false positive rate (FPR) and true positive rate (TPR) as evaluation indicators. True positive (TP) is the number of cells identified as NegLFC by the method and the same as ground truth. False positive (FP) is the number of cells identified as NegLFC by the method but ground truth is PosLFC. False negative (FN) is the number of cells identified as PosLFC by the method but ground truth is NegLFC. True negative (TN) is the number of cells identified as PosLFC by the method and the same as ground truth. Then we use FPR and TPR to evaluate the effectiveness of all methods, defined as follows:

$$FPR = \frac{FP}{FP + TN}$$

$$TPR = \frac{TP}{TP + FN}$$

Text S6. Simulation of batch effects

To evaluate the robustness of reDA, we introduced artificial batch effects into the simulated data. Cells were partitioned into two batches, and a Gaussian random vector was generated and incorporated into the principal component profile of all cells in the same batch. By varying the 'norm_sd' parameter across five simulated datasets, we generated a gradient of artificial batch effects with intensities ranging from 0 to 1.5.

Text S7. The overlap between differentially accessible regions and genetic loci

In order to assess the potential association between chromatin accessibility changes and genetic loci related to colorectal cancer, we used the gwasrapidd R package (Magno and Maia, 2020) to extract colorectal cancer-associated genetic loci with the p-value threshold of 1×10^{-6} from the GWAS Catalog database (Sollis et al. 2023). Subsequently, we examined overlaps between the differentially accessible regions (DARs) identified in four disease-associated cell subsets and these GWAS-identified genetic loci. The overlap analysis was performed using the findOverlaps() function. We identified overlaps between DARs and genetic loci across four cell subsets: cancer-associated fibroblasts (rs73376930, rs2293582, rs2293581, rs79207432, rs1919364, rs4807543, rs209142, rs10936599), exhausted T cells (rs12822620), regulatory T cells (rs12822620, rs4807543, rs10936599, rs11064437, rs12979278), and enterocytes (rs1810502, rs1810501, rs6067449, rs4444073, rs2250430, rs17572109, rs2250430, rs78368589, rs77776598, rs10409772, rs2236200, rs1815949, rs1362126). These findings further confirm the relevance of the identified chromatin accessibility changes to colorectal cancer-associated genetic variations.

Supplementary Figures

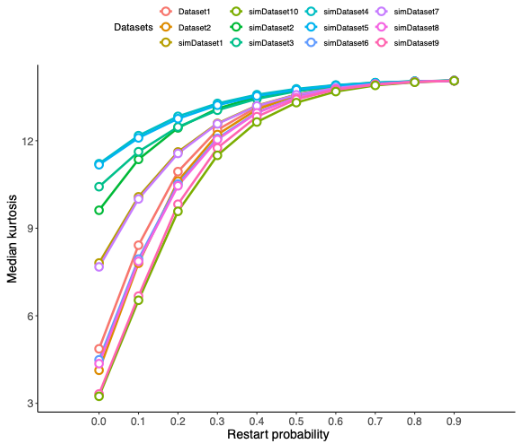

Figure S1 Median kurtosis increases with the restart probability on two real and ten simulated scATAC-seq datasets with ground truth labels (Supplementary Table S5).

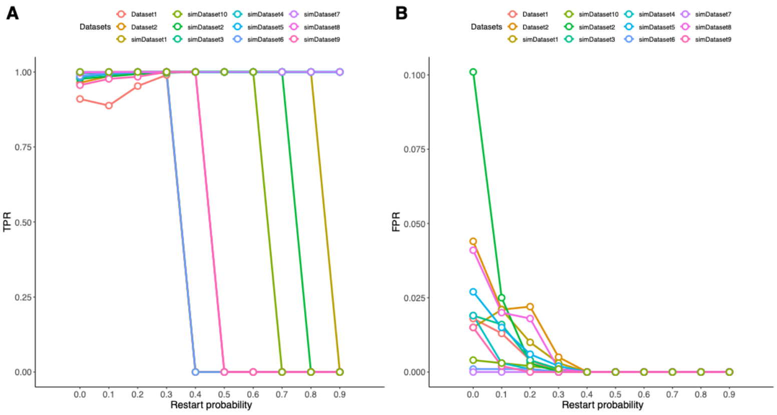

Figure S2 TPR and FPR changes with increasing restart probability on two real and ten simulated scATAC-seq datasets with ground truth labels (Supplementary Table S6). When the restart probability is 0, the restart probability is not used.

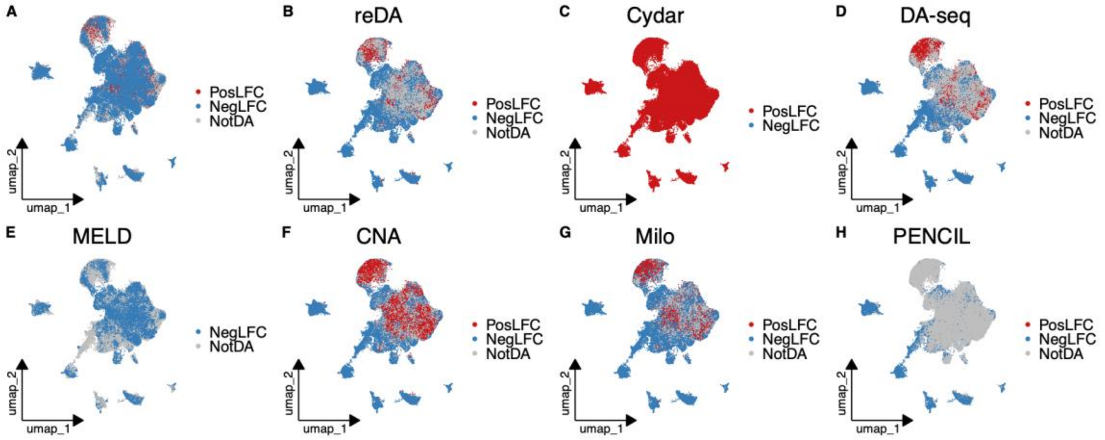

Figure S3 The UMAP visualization of the real scATAC-seq Dataset1 with ground truth labels. Cells are colored by ground truth labels (A) and the results identified by different methods (B-H).

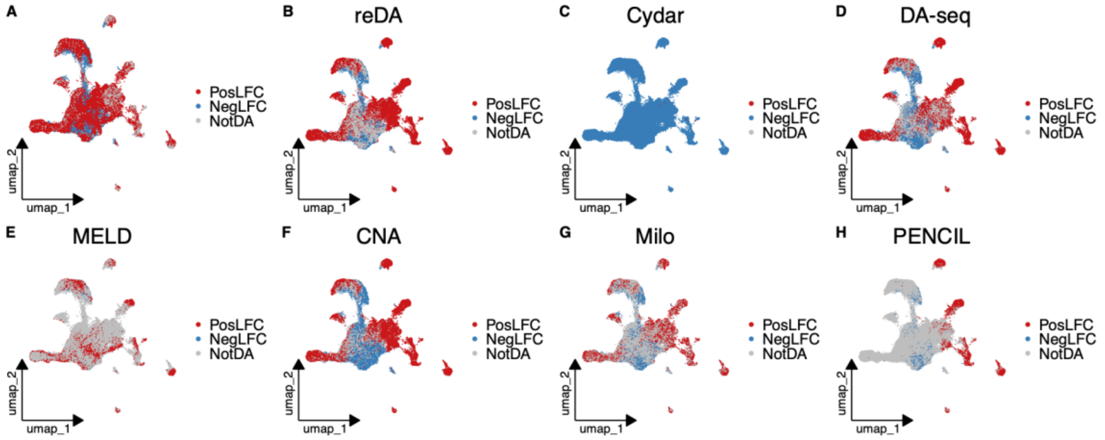

Figure S4 The UMAP visualization of the real scATAC-seq Dataset2 with ground truth labels. Cells are colored by ground truth labels (A) and the results identified by different methods (B-H).

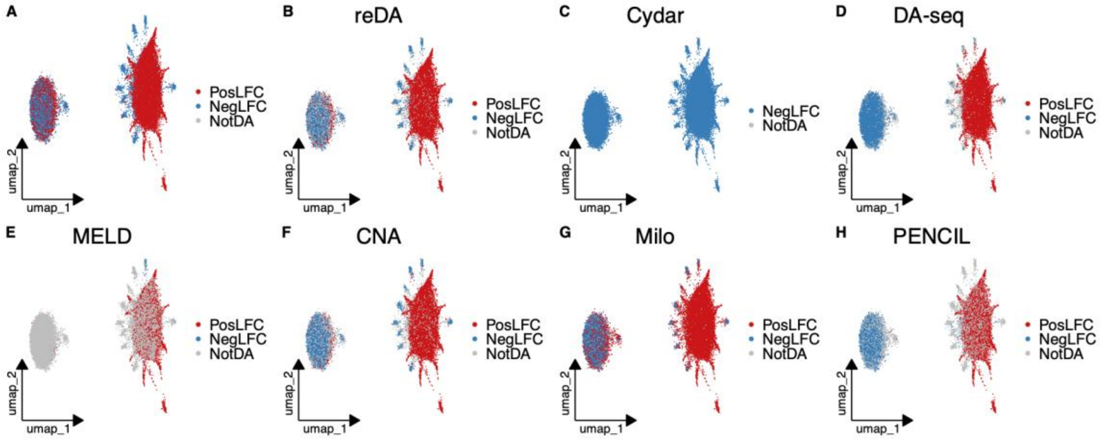

Figure S5 The UMAP visualization of the simulated scATAC-seq simDataset1 with ground truth labels. Cells are colored by ground truth labels (A) and the results identified by different methods (B-H).

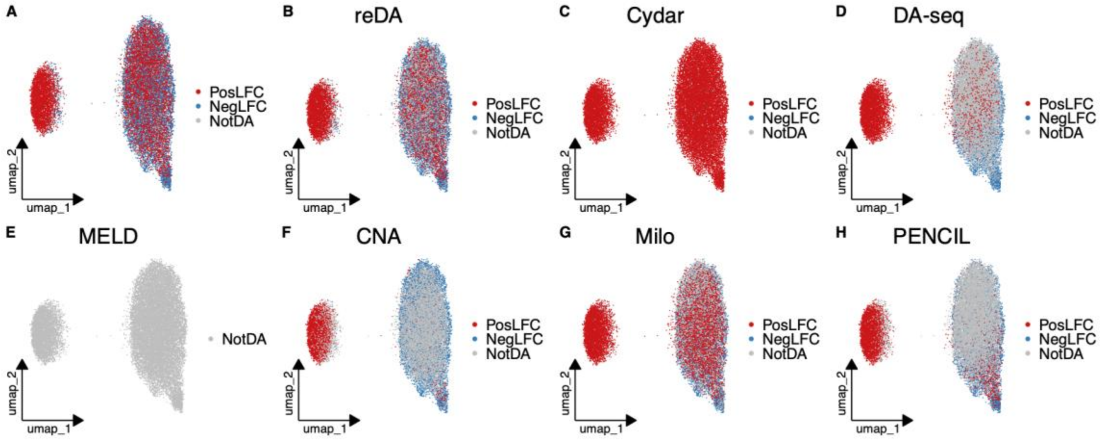

Figure S6 The UMAP visualization of the simulated scATAC-seq simDataset2 with ground truth labels. Cells are colored by ground truth labels (A) and the results identified by different methods (B-H).

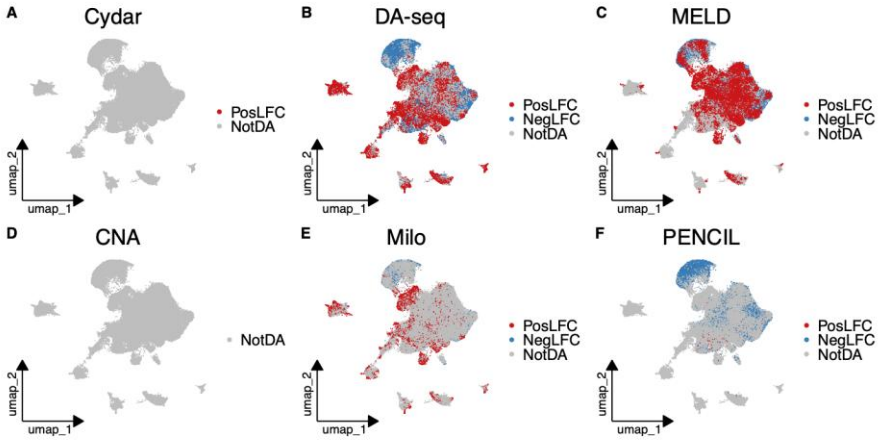

Figure S7 The UMAP visualization of the cells from CRC and FAP patient samples. Cells are colored by the results identified by different methods (A-F).

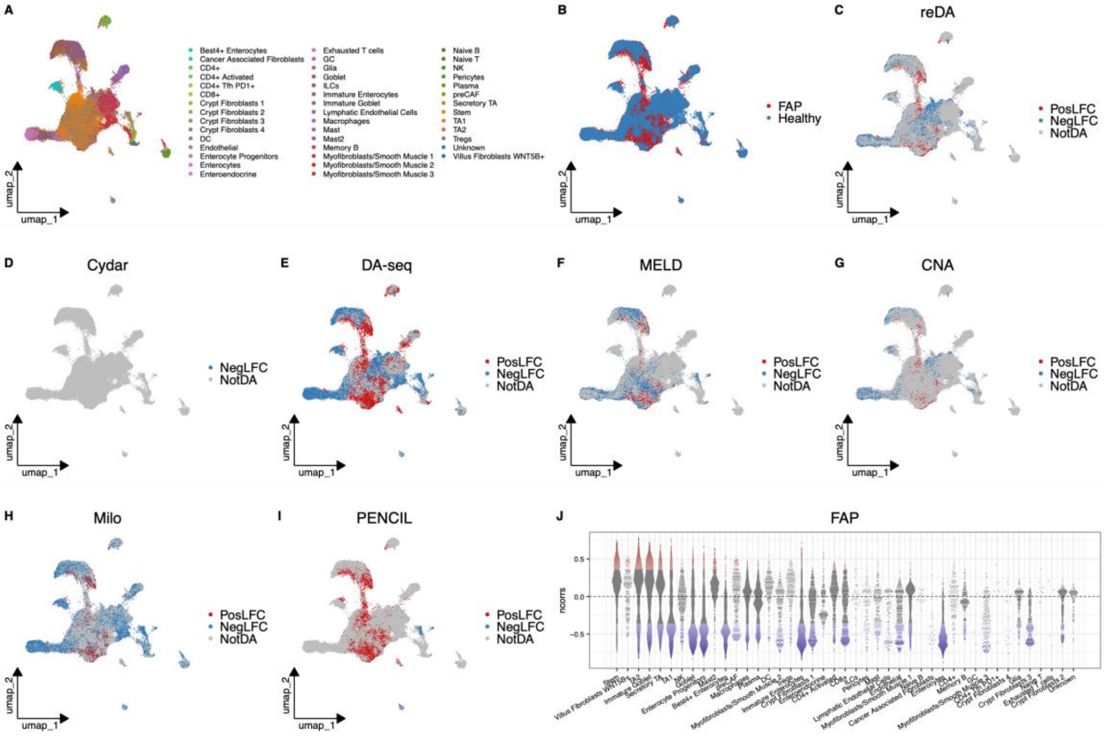

Figure S8 A-I, UMAP embedding of the cells from FAP and healthy patient samples. Cells are colored by cell types (A), sample conditions (B), and the results identified by different methods (C-I). **J,** Beeswarm plot showing the distribution of neighborhood coefficients from different cell types. Differential abundance cells at FDR 5% are colored.

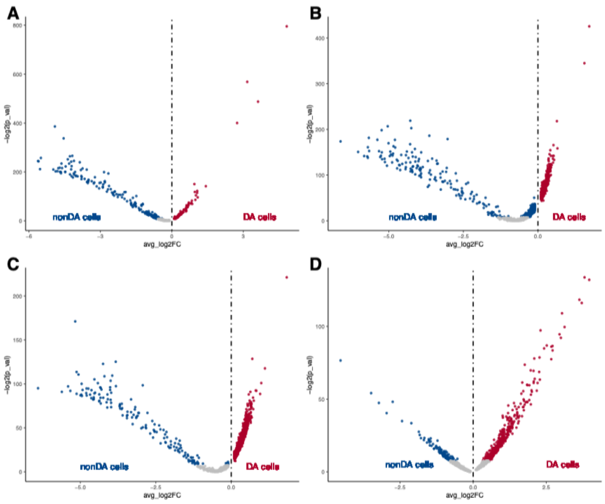

Figure S9 Volcano plots of peaks identified in the four cell subsets and their background cell populations. CAFs (A), Exhausted T cells (B), Tregs (C), and Enterocytes (D).

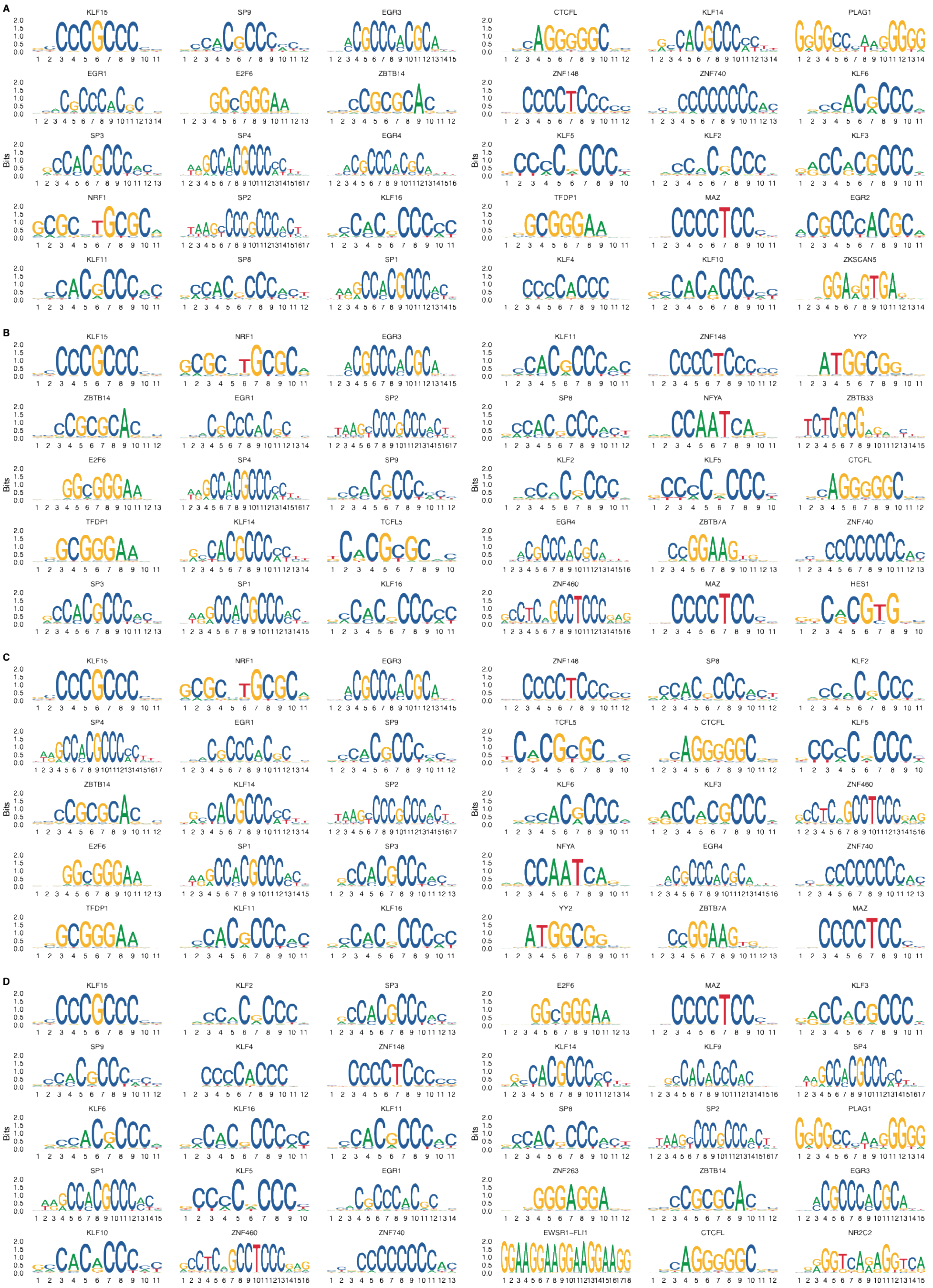

Figure S10 the transcription factor motifs are enriched on CAFs (A), Exhausted T cells (B), Tregs (C), and Enterocytes (D).

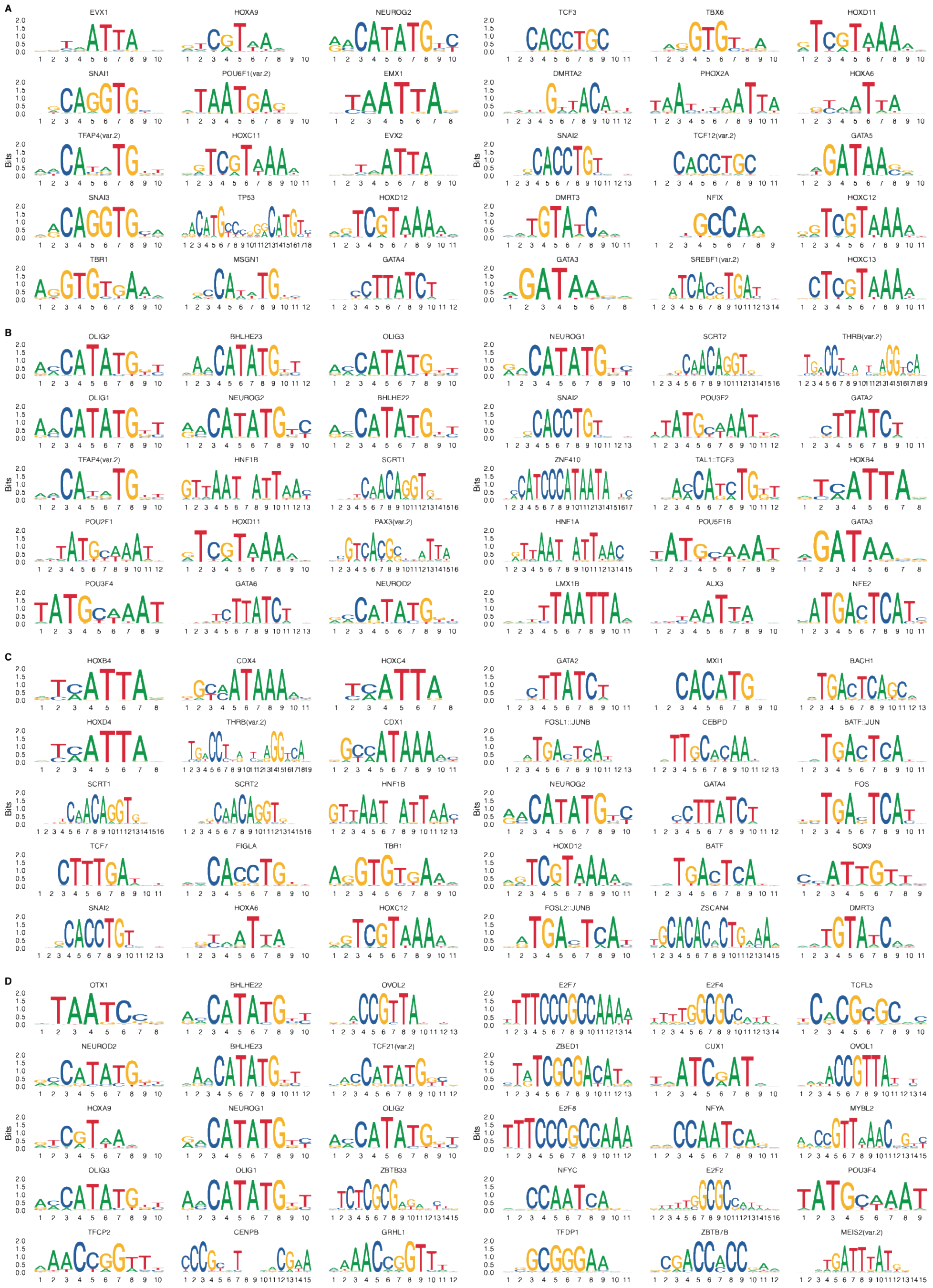

Figure S11 the transcription factor motifs are enriched on the background cells of CAFs (A), Exhausted T cells (B), Tregs (C), and Enterocytes (D).

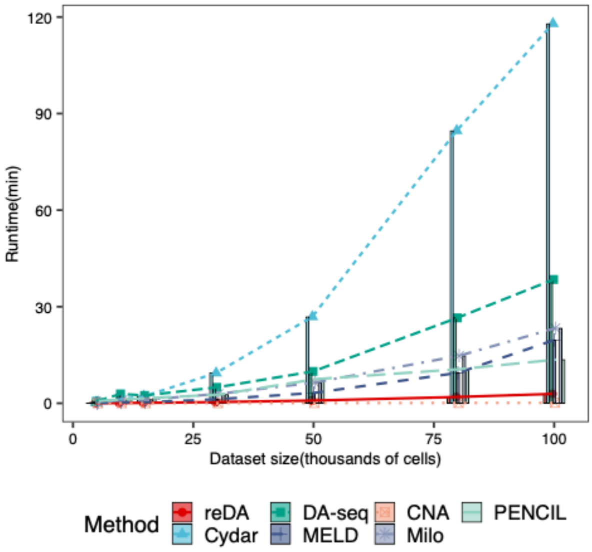

Figure S12 Runtime of the reDA and other six existing methods on the single-cell ATAC-seq datasets varies with the increasing number of cells (5k, 10k, 15k, 30k, 50k, 80k, 100k) (Supplementary Table S7).

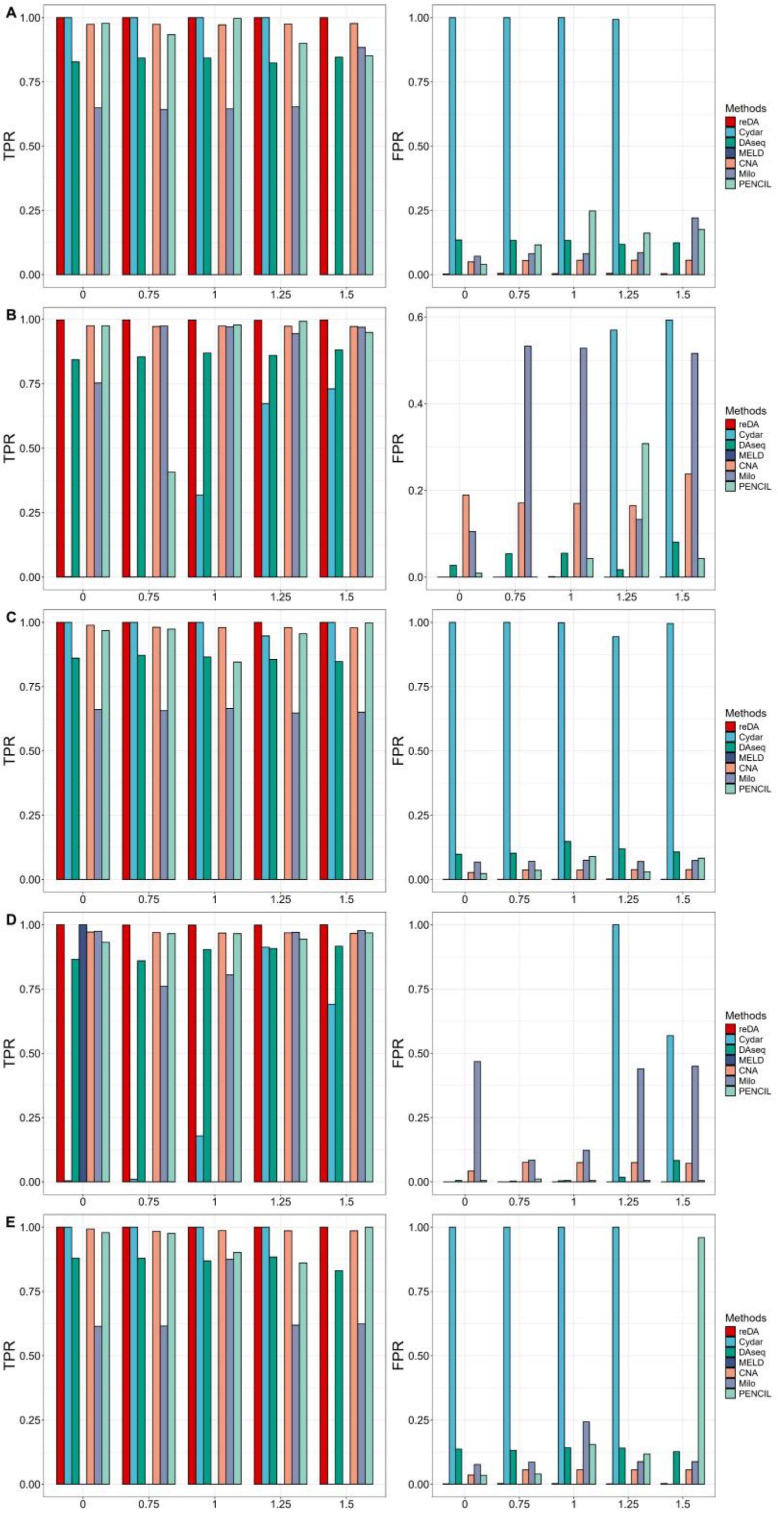

Figure S13 A-E TPR and FPR of reDA and comparison methods on five simulated datasets (A: simDataset1, B: simDataset1, C: simDataset1, D: simDataset1, E: simDataset5) with ground truth labels and varying degrees of artificially introduced batch effects (Supplementary Table S8). 0 indicates the absence of batch effects.

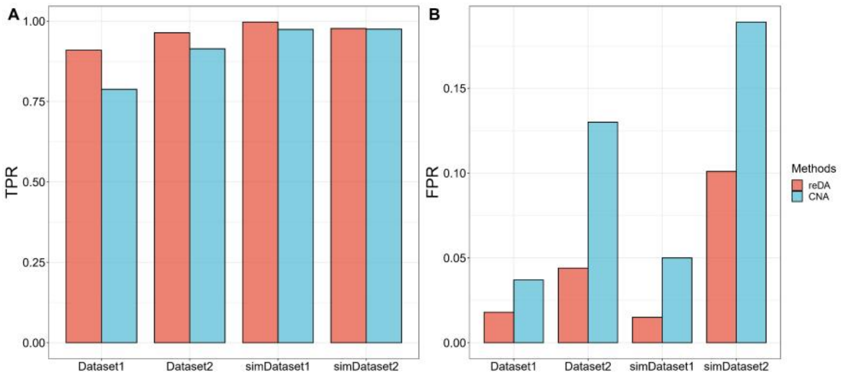

Figure S14 TPR and FPR of reDA (without the use of restart probability) and CNA on real and simulated data with ground truth labels (Supplementary Table S9).

Reference

Magno, R. and Maia, A.T. Gwasrapidd: an R package to query, download and wrangle GWAS catalog data. *Bioinformatics* 2020;36(2):649-650.

Sollis, E., *et al*. The NHGRI-EBI GWAS Catalog: knowledgebase and deposition resource. *Nucleic Acids Res* 2023; 51(D1): D977-D985.
